# Polymorphisms and AR: A Systematic Review and Meta-Analyses

**DOI:** 10.3389/fgene.2022.899923

**Published:** 2022-07-01

**Authors:** Feng Xiang, Zhen Zeng, Lu Wang, Ye Peng Yang, Qin Xiu Zhang

**Affiliations:** ^1^ Clinical Medical College, Chengdu University of Traditional Chinese Medicine, Chengdu, China; ^2^ School of Medical and Life Sciences, Chengdu University of Traditional Chinese Medicine, Chengdu, China

**Keywords:** allergic rhinitis, polymorphism, meta-analysis, IL-13, CTLA-4, IL-4R, ACE

## Abstract

**Background:** Allergic rhinitis (AR) is an especially common disorder associated with both environmental and genetic factors, and a lot of researchers have attempted to find polymorphisms which predisposed to the disease. We conducted a meta-analysis of the most frequently researched polymorphisms to find those genes which may be susceptible to AR and then may be of value in diagnosis.

**Methods:** Pubmed and China National Knowledge Infrastructure (CNKI) databases were searched to screen out eligible studies focusing on the correlation between polymorphisms and AR susceptibility, and then polymorphisms cited in at least 3 studies were selected.

**Results:** The 142 papers originally selected cited 78 genes. Twelve genes (coinciding with 23 polymorphisms) were reported in more than three papers. Twenty-three polymorphisms were involved in the meta-analysis. Among the 23 polymorphisms, only 4 were found to be related to the risk of AR: IL-13 rs20541, CTLA-4 rs11571302, IL-4R RS1801275 and ACE (I/D). The remaining 19 of the 23 polymorphisms were not associated with AR.

**Conclusion:** We found polymorphisms that could be used for AR diagnosing and those that were unrelated to AR. This may be the first step in detecting polymorphic combinations susceptible to AR (IL-13 RS20541, CTLA-4 RS11571302, IL-4R RS1801275 and ACE (I/D). In addition, our results may improve AR diagnosis and contribute to the intensive study of AR.

## 1 Introduction

Allergic rhinitis (AR) is a chronic inflammatory disorder of the nasal mucosa mediated by allergic hypersensitivity responses to environmental allergens (Greiner et al., 2011). As reported, 10%–20% of the world’s population suffers from AR (Brożek et al., 2010), which has become a global health problem. However, the underlying cause for AR is poorly understood and prevention of the disease is impossible. AR is a complex heterogeneous disease involving genetic and environment factors (Barnes, 2000). Studies of twins have shown that genetic factors play a significant role in the pathogenesis of allergic disease (Khan et al., 2018), and individuals with a family history of allergic disease are more likely to develop allergic symptoms (Sih and Mion, 2010). Single nucleotide polymorphisms (SNPs) are considered to be an extremely crucial genetic factor for allergic diseases (Suzuki et al., 2016; Ashley et al., 2017) which can upregulate or downregulate the susceptibility of AR (Black et al., 2009). Tang et al. (2020) elucidated that IL13 rs20541 SNP may contribute to the susceptibility to AR and increase the risk of AR in Asian population. Besides, Qian et al. (2010) demonstrated that the A allele of rs11466651 in TLR10 will reduce the susceptibility of AR, which mean that the polymorphism protects against AR. Furthermore, the association of SNPs with AR also exists controversial. For example, some researchers have found that IL-4 rs2243250 allele T could increase or decrease the risk of AR (Lu et al., 2011; Movahedi et al., 2013; Shirkani et al., 2019). In contrast, others believed that allele T is not related to an increased prevalence of AR (Valatabar et al., 2020).

To solve the limitations of individual researches, a comprehensive evaluation of the relationship between SNPs and vulnerability to AR is required in synthesized studies. The purpose of this study was, first, to provide a detailed review of the SNPs researched in AR, and second, to evaluate the correlation between the most frequently reported SNPs and the susceptibility to AR by meta-analysis, so as to further understand the molecular mechanism of this disease and provide underlying therapeutic targets or approaches for the reuse of existing drugs.

## 2 Methods

### 2.1 Search Strategy

We performed a exhaustive literature search on PubMed and CNKI, with the search strategies being (“allergic rhinitis” or “AR”) and (“mutation” or “polymorphism”). The detailed search strategy is supplied in Supplementary Table S1. There were no restrictions on language or date of publication. The literature search was finally updated on 11 February 2022. Eligibility for this publication was independently evaluated by two researchers (FX and LW). For purpose of reviewing the literature as widely and efficiently as possible, we also reviewed references of relevant studies to supplement the valuable literature. In the event of uncertainty, the full text was acquired and any disagreement was resolved by the reviewer’s agreement.

### 2.2 Study Selection

In order to gain the credibility of our results and restrict the amount of meta-analyses completed, only the genes and polymorphisms reported in at least three diverse studies were considered in the analysis.

For meta-analysis, studies must conform to the following inclusion standard: 1) original case-control studies of AR; 2) An association between AR and at least one polymorphism was reported and sufficient data were available to calculate odds ratio (OR) and corresponding 95% CI; 3) Genotype frequency did not deviate from Hardy-Weinberg equilibrium (HWE) significantly; 4) Based on the symptoms and allergen tests or confirmed histologically, people were diagnosed as AR. Duplicate researches and researches with overlapping data were excluded. We also excluded retrospective studies, systematic reviews, and meta-analyses.

Exclusion criteria were as follows: 1) not a case-control study; 2) irrelevant to SNPs and AR risk; 3) Lack of detailed data.

### 2.3 Data Extraction

One investigator (FX) extracted the data from every eligible study, which was checked by the second investigator (LW). Divergences were settled by consultation with the third researcher (YP). For each study, the following data were collected: the title, the authors, the journal, the year of publication, the country of study team, the sample capacity, and the method of diagnose.

### 2.4 Statistical Analyses

After data extraction, meta-analyses for polymorphisms in at least three publications were performed. The RevMan 5.3 software was employed to complete all statistical analyses. The degree of association between AR and interested polymorphism was evaluated. A random-effect OR fixed-effect model was applied to completed meta-analysis through merging OR (95% CI) values of the involved publications. Before merging the publications, heterogeneity needs to be found correctly and effectively, that is, heterogeneity test. I2 test is the heterogeneity evaluation index proposed by Higgins et al. (2003). When I2 = 0, it indicates that no heterogeneity is observed, and the higher I2 value, the greater heterogeneity. The selection of model lies with the heterogeneity of inter-studies. When there was no evidence of heterogeneity, the fixed-effect model was selected (I2 < 50%, *p* > 0.05) (Li et al., 2018; Li et al., 2019; Li et al., 2020). If not, a random-effect model was used. A value of OR greater than 1 with a CI lower limit greater than 1 and *p* < 0.05 is believed to be statistically significant, or less than 1 with the upper CI boundary less than 1.

The publication bias was judged intuitively by drawing a funnel plot. The funnel plot symmetric, no publication bias was found; otherwise, publication bias existed. Besides, publication bias was quantitatively estimated by the Egger’s Test. For every polymorphism involved in this research, articles search was conducted to identify prior meta-analyses. And our results were compared with the prior results to identify potential records.

## 3 Results

### 3.1 Search Methods and Data Screening

**FIGURE 1:**
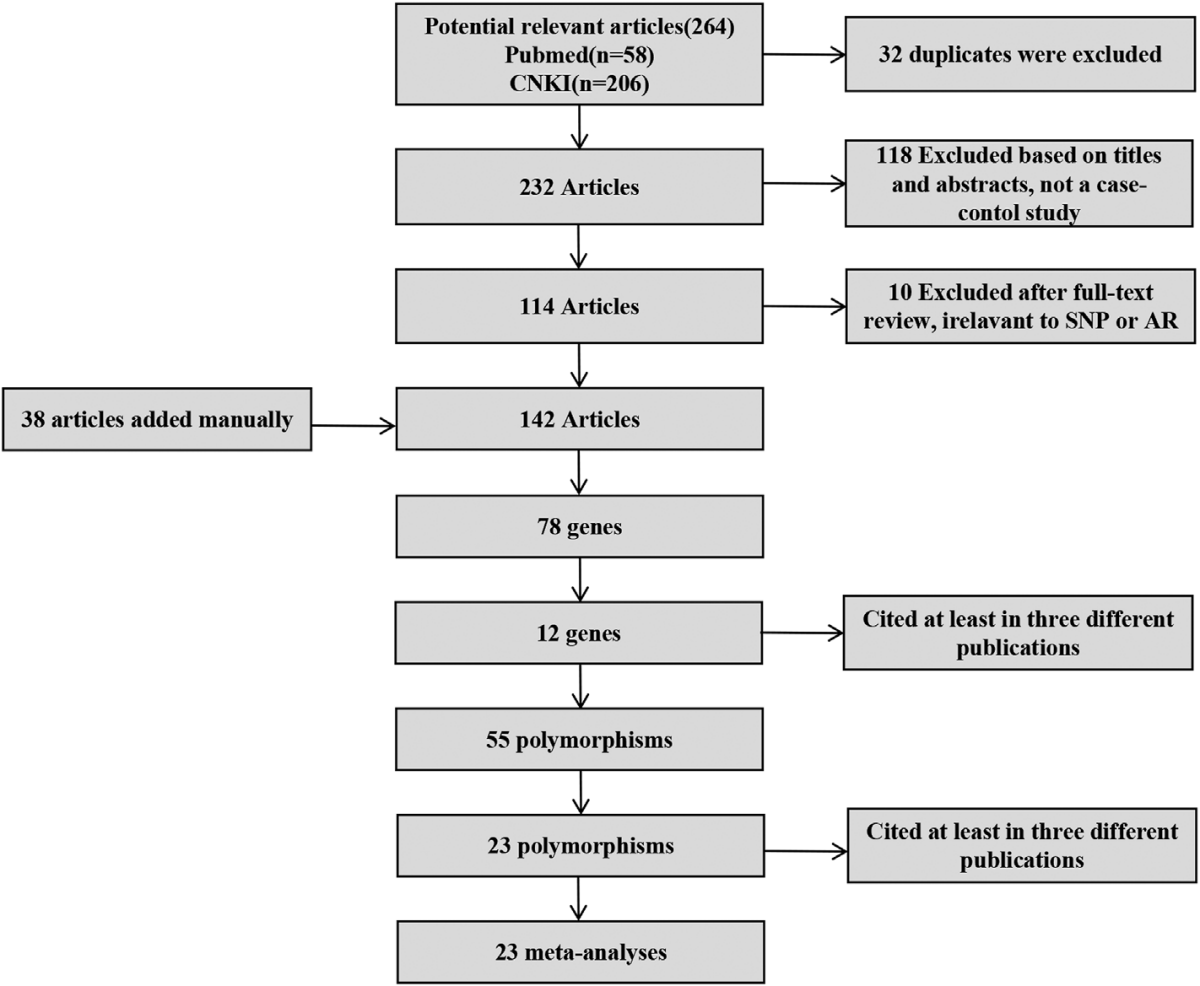
Screening of polymorphisms for meta-analysis.

After screening (Figure 1), a total of 23 polymorphisms that had been reported in at least 3 literatures were identified. 142 papers (Supplementary Table S1) reported 78 diverse genes, and 12 of those were studied in at least three publications. 55 polymorphisms were identified totally in the present study (Supplementary Table S2). Then, a systematic review were completed for each of the 23 screening polymorphisms, which were studied in more than three publications (Table 1). The number of publications including every polymorphism, the degree of heterogeneity, the amount of patients and controls, the OR [95% CI] and the application of a random-effect or fixed-effect model are showed in Table 1.

**TABLE 1:** The primary results of the meta-analyses in exploring the relationship between gene polymorphisms and AR.

Gene	Polymorphisms	Minor allele	Total Studies	Number of patients		Model	heterogenity test	Fixed or random effects model	OR	CI	*p*.value	Funnel plot asymmetry linear regression test	
				AR	Control	allelic/genomic	*p*.value					t	*p*.value
TNFα	rs1800629−308G/A	A	5	1021	887	Allelic	0.004	Random	1.56	[1.00; 2.42]	0.05	−0.4	0.717
TGFβ1	rs1800469−509C/T	**C**	4	560	519	Allelic	<0.001	Random	0.89	[0.37; 2.12]	0.79	−0.51	0.659
IL-13	rs1800925−1112C>T	T	5	984	1284	Allelic	0.35	Fixed	0.99	[0.85; 1.17]	0.94	0.01	0.994
	rs20541+2044G>A	A	12	2924	2782	Allelic	0.05	Fixed	1.22	[1.12; 1.33]	<0.001	1.19	0.262
CTLA-4	rs3087243	A	3	1039	1142	Allelic	0.54	Fixed	1.06	[0.93; 1.20]	0.38	−3.7	0.168
	rs231725	**G**	3	1041	1134	Allelic	0.06	Random	0.81	[0.63; 1.05]	0.12	−4.81	0.13
	rs11571302	A	3	1040	1143	Allelic	0.17	Fixed	1.41	[1.24; 1.60]	<0.001	−10.67	0.059
	rs11571315	**G**	3	1041	1144	Allelic	0.14	Fixed	1.00	[0.89; 1.55]	0.97	4.29	0.146
IL-4	rs2243250−590C>T	T	8	1660	1807	Allelic	<0.001	Random	1.37	[0.95; 1.98]	1.37	0.71	0.506
	rs2227284T2979G	G	3	657	963	Allelic	<0.001	Random	1.56	[0.64; 3.80]	0.33	1.64	0.349
	rs2070874C−33T	**C**	3	597	962	Allelic	0.0003	Random	0.68	[0.34; 1.37]	0.28	−2.24	0.267
CD14	rs2569190−159C/T	T	10	1787	1820	Allelic	0.0007	Random	0.93	[0.78; 1.12]	0.46	1.71	0.126
FOXP3	rs3761548−3279A>C	**A**	5	935	925	Allelic	0.005	Random	0.89	[0.65; 1.20]	0.44	0.12	0.910
	rs2232365−924A>C	C	4	796	763	Allelic	0.29	Fixed	0.97	[0.83; 1.13]	0.69	0.82	0.498
IL-18	rs1946518−607C/A	A	4	1362	833	Allelic	0.28	Fixed	1.02	[0.90; 1.16]	0.74	2.96	0.098
	rs187238−137G/C	C	3	1202	662	Allelic	0.73	Fixed	0.91	[0.78; 1.06]	0.22	2.22	0.269
	rs4988359/rs360721 C133/140G	G	3	1202	662	Allelic	0.41	Fixed	0.89	[0.77; 1.04]	0.14	10.68	0.059
Tim-3	rs10515746−574G/T	T	4	571	691	Allelic	0.14	Fixed	1.21	[0.78; 1.88]	0.40	2.84	0.105
IL-4R	rs1801275Q576R//Gln551Arg	R	9	924	920	Allelic	0.003	Random	1.48	[1.07; 2.05]	0.02	2.62	0.034*
	rs1805010 I50V	V	3	179	197	Allelic	0.75	Fixed	0.84	[0.63; 1.13]	0.25	0.51	0.699
ACE	I/D	D	5	561	603	Allelic	0.03	Random	1.47	[1.09; 1.97]	0.01	1.25	0.301
TAP1	333V/I	**V**	4	231	270	Allelic	<0.001	Random	1.26	[0.50; 3.15]	0.62	−0.57	0.627
TAP1	637G/D	**G**	4	204	270	Allelic	<0.001	Random	0.72	[0.31; 1.69]	0.45	0.00	0.999

### 3.2 Meta-Analysis Results

#### TNF_ rs1800629 (-308G/A)

**FIGURE 2:**
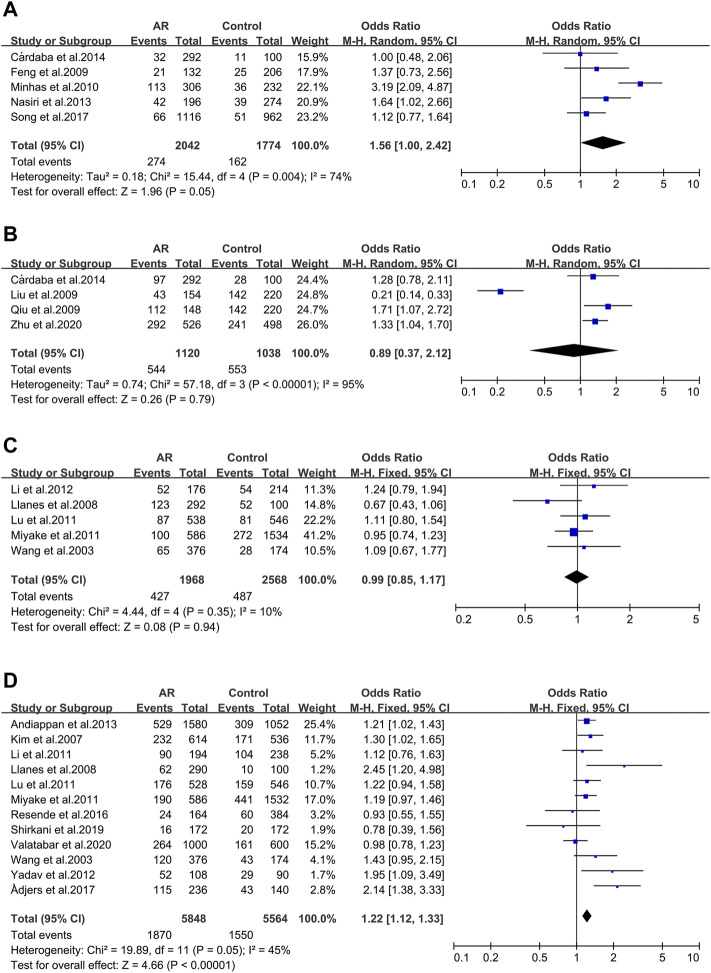
Forest plots for TNFα, TGFβ1 and IL-13 polymorphisms. **(A)** TNFα(rs1800629): A allele; **(B)** TGFβ1 (rs1800469): allele G; **(C)** IL-13 (rs1800925): allele T; **(D)** IL-13 (rs20541): allele A.

Five studies evaluated six tumor necrosis factor (TNF) variants, of which only TNF RS1800629 was considered in our work. Five literatures were included in this meta-analysis (30/59/102/103/104). We didn’t observe significant associations (OR 1.56; 95% CI 1.00-2.42) applying a random-effect model (Figure 2A), and publication bias wasn’t observed as well (*t* = −0.4; *p* = 0.717) (Supplementary Figure S1A).

#### TGFβ1 _ rs1800469 (-509C/T)

Four studies of the Transforming growth factor β1 (TGFβ1) rs1800469 polymorphism were included (32/65/104/141). We didn’t observe significant associations (OR 0.89; 95% CI 0.37–2.12) applying a fixed-effect model (Figure 2B) and publication bias wasn’t observed as well (*t* = −0.51; *p* = 0.659) (Supplementary Figure S1B).

#### 3.2.1 The IL-13 Gene

Thirteen full-text articles on Interleukin-13(IL-13) SNPs of interest were selected. Two genetic variants of IL-13 were analyzed: rs1800925 (Figure 2C), rs20541 (Figure 2D).

IL-13 _ rs1800925 (-1112C > T/1055). IL-13 _ rs1800925 (-1112C > T/1055) was considered in five studies (41/100/107/117/118) and meta-analysis was completed on them. We didn’t observe significant associations (OR 0.99; 95% CI 0.85–1.17) in a fixed-effect model (Figure 2C), and publication bias wasn’t seen as well (*t* = 0.01; *p* = 0.994) (Supplementary Figure S1C).

IL-13_rs20541 (130/ + 2044G > A). Twelve studies dealing with the IL-13_rs20541 A allele were considered (42/100/105/107/108/110/111/113/116/117/118/119) for the meta-analysis. An association was observed (OR 1.22; 95% CI 1.12–1.33) applying a fixed-effect model (Figure 2D) without publication bias (*t* = 1.19; *p* = 0.262) (Supplementary Figure S1D).

#### 3.2.2 The CTLA-4 Gene

**FIGURE 3:**
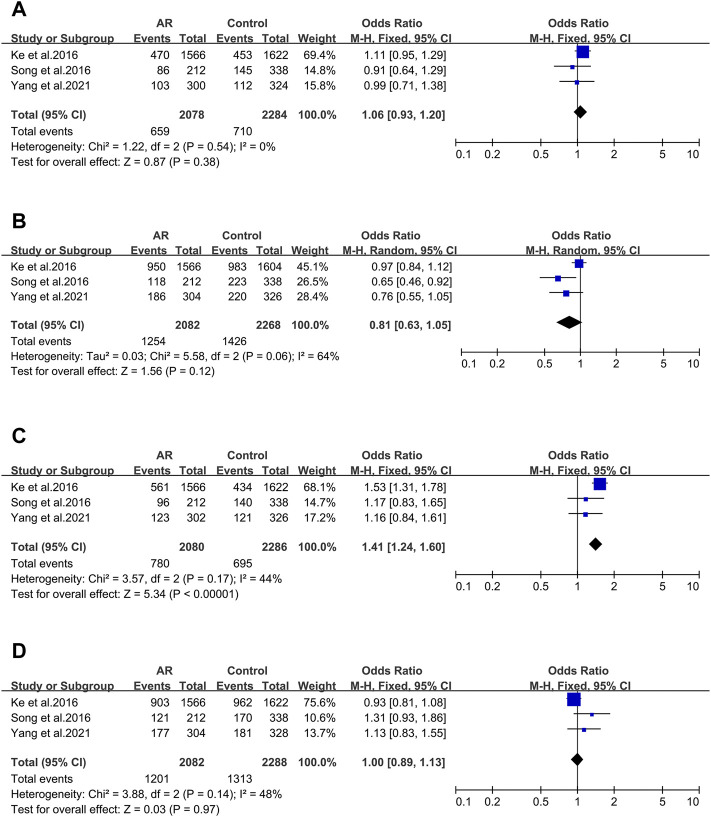
Forest plots for CTLA-4 polymorphism. **(A)** CTLA-4 (rs3087243); **(B)** CTLA-4 (rs231725); **(D)** CTLA-4 (rs11571302); **(D)** CTLA-4 (rs11571315).

After screening, four of the nine main allelic mutants of Cytotoxic T lymphocyte-associated antigen 4 (CTLA-4) were analyzed: rs3087243 (Figure 3A), rs231725 (Figure 3B), rs11571302 (Figure 3C) and rs11571315 (Figure 3D), and only rs11571302 was significantly associated with AR.

CTLA-4 _ rs3087243. Three studies were included (12/94/106). We didn’t observe significant associations (OR 1.06; 95% CI 0.93–1.20) applying a fixed-effect model (Figure 3A). And asymmetry wasn’t seen in the funnel plot test for bias as well (*t* = −3.7; *p* = 0.168) (Supplementary Figure S1E).

CTLA-4 _ rs231725. Three studies (12/94/106) were involved in the meta-analysis. We didn’t find significant associations (OR 0.81; 95% CI 0.63–1.05) applying a random-effect model (Figure 3B), and publication bias wasn’t seen as well (*t* = -4.81; *p* = 0.13) (Supplementary Figure S2F).

CTLA-4 _ rs11571302. CTLA-4 variant (rs11571302) was considered in three studies (12/94/106). When we considered the A allele as the risk allele, a correlation was found (OR 1.41; 95% CI 1.24–1.60) in a fixed-effect model (Figure 3C) with no publication bias (*t* = −10.67; *p* = 0.059) (Supplementary Figure S2G).

#### CTLA-4_rs11571315

Three studies were considered (12/94/106). We didn’t observe significant associations (OR 1.00; 95% CI 0.89–1.55) in a fixed-effect model (Figure 3D), and publication bias wasn’t seen as well (*t* = 4.29; *p* = 0.146) (Supplementary Figure S2H).

#### 3.2.3 The IL-4 Gene

In the 8 studies of AR and Interleukin-4(IL-4) polymorphisms five IL-4 variants were reported. Only three of them were considered.

**FIGURE 4:**
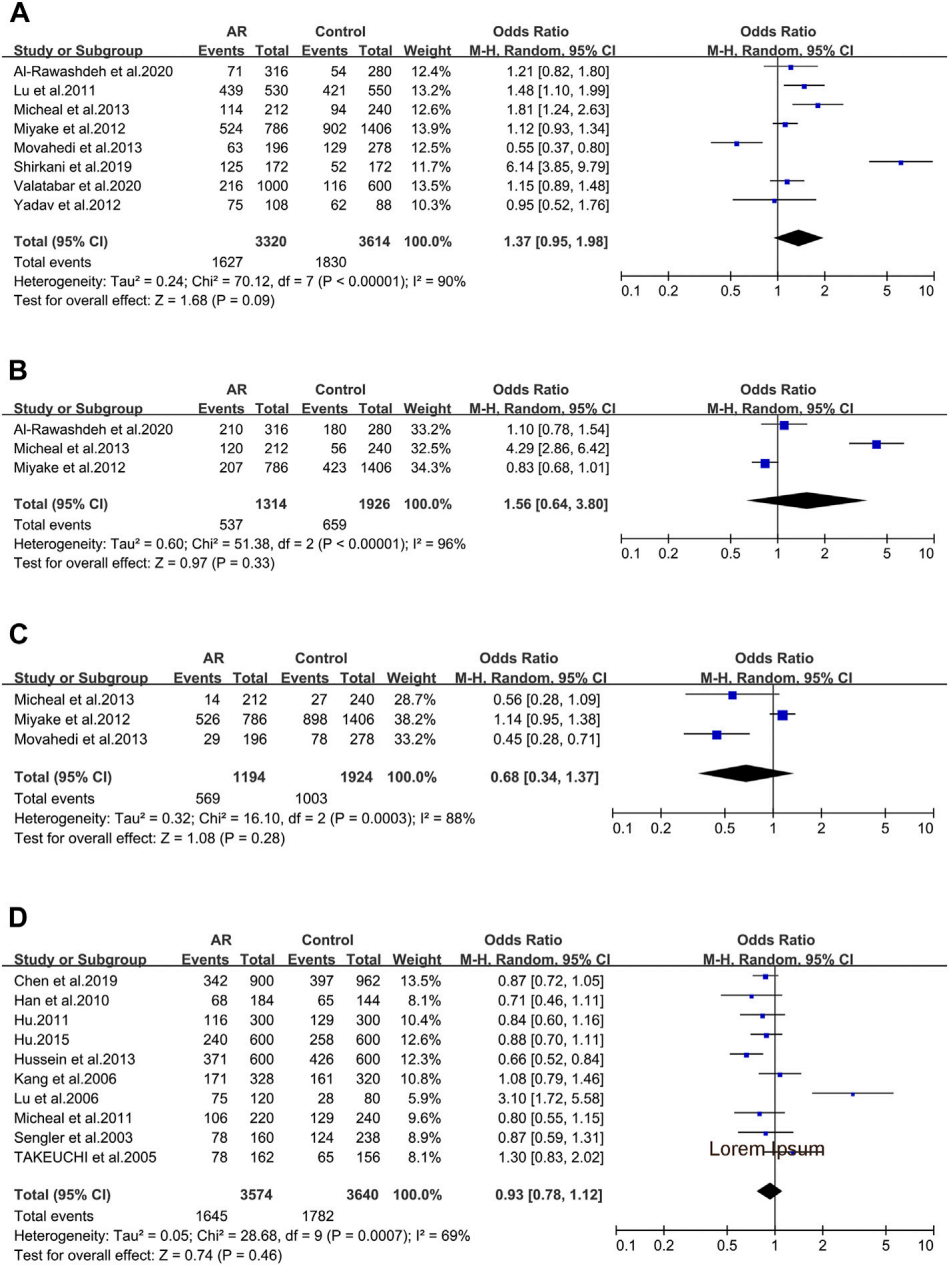
Forest plots for IL-4 and CD14 polymorphisms. **(A)** IL-4 (rs2243250): T allele; **(B)** IL-4 (rs2227284): allele G; **(C)** IL-4 (rs2070874): allele T; **(D)** CD14 (rs2569190): allele T.

IL-4 _ rs2243250 (−590C > T/589). IL-4 _ rs2243250 was considered in eight studies (107/108/109/110/112/113/114/115). When we considered the T allele as the risk allele, associations were not identified (OR 1.37; 95% CI 0.95–1.98) in a random-effect model (Figure 4A), and publication bias wasn’t seen as well (*t* = 0.71; *p* = 0.506) (Supplementary Figure S2A).

IL-4 _ rs2227284 (T2979G). Three studies was also considered (112/114/115) and meta-analysis was completed on these studies. We didn’t find significant associations (OR 1.56; 95% CI 0.64–3.80) in a random-effect model (Figure 4B), and publication bias wasn’t seen as well (*t* = 1.64; *p* = 0.349) (Supplementary Figure S2B).

IL-4 _ rs2070874(C-33T). Three studies (109/112/114) were included in this meta-analysis. We didn’t observe significant associations (OR 0.68; 95% CI 0.34–1.37) applying a random-effect model (Figure 4C), and publication bias wasn’t detected as well (*t* = −2.24; *p* = 0.267) (Supplementary Figure S2C).

When considering the 10 studies of CD14 variants and AR, solely the rs2569190 polymorphism conformed to the inclusion standard. The ten publications were pooled (5/7/47/63/91/120/121/122/123/124), and we didn’t find significant associations (OR 0.93 95% IC0.78–1.12) applying a random-effect model (Figure 4D), as well as the publication bias (*t* = 1.71; *p* = 0.126) (Supplementary Figure S2D).

#### 3.2.4 The FOXP3 Gene

**FIGURE 5:**
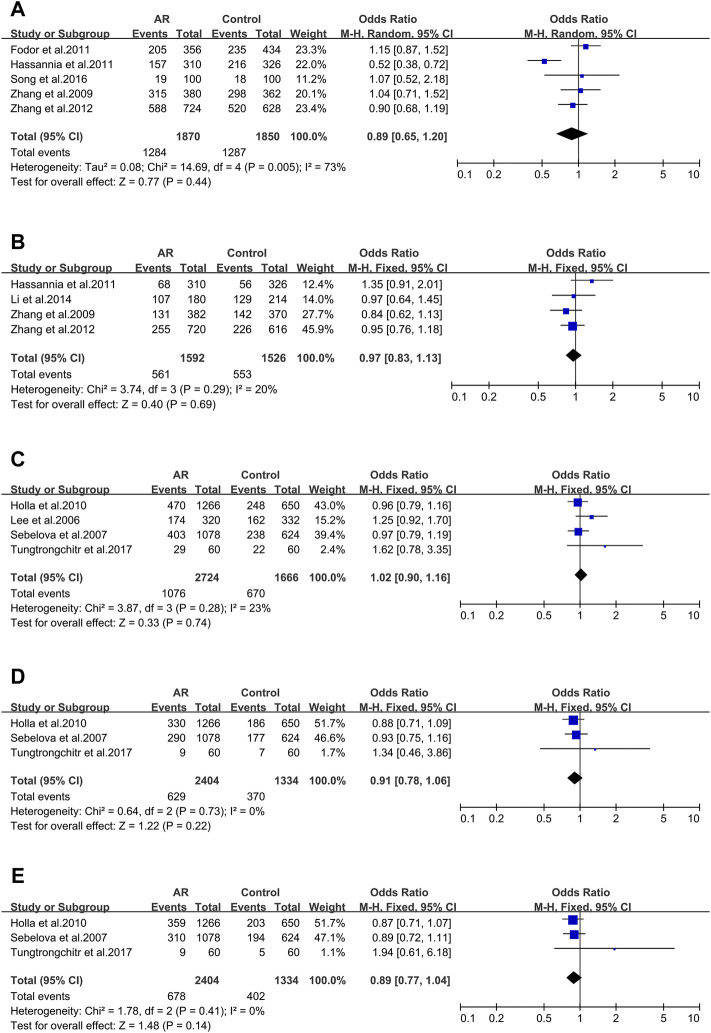
Forest plots for FOXP3 and IL-18 polymorphisms. **(A)** FOXP3 (rs3761548): C allele; **(B)** FOXP3 (rs2232365): C allele; **(C)** IL-18 rs1946518): allele A; **(D)** IL-18 (rs187238): allele C; **(E)** IL-18 (rs4988359): allele G.

Two of the four primary genetic variants of forkhead box P3(FOXP3) were analyzed: rs3761548 (Figure 5A) and rs2232365 (Figure 5B), and none was significantly associated with AR.

FOXP3_ rs3761548 (−3279A > C). Five studies (50/84/125/126/127) were considered for meta-analysis. Association wasn’t identified (OR 0.89; 95% CI 0.65–1.20) employing the random-effect model (Figure 5A) and publication bias wasn’t found (*t* = 0.12; *p* = 0.910) (Supplementary Figure S2E).

FOXP3_ rs2232365 (−924A > C). Four studies (125/126/127/128) were involved (Supplementary Figure S26A). We didn’t find significant associations (OR 0.97; 95% CI 0.83–1.13) in a fixed-effect model (Figure 5B), and publication bias wasn’t found as well (*t* = 0.82; *p* = 0.498) (Supplementary Figure S2F).

#### 3.2.5 The IL-18 Gene

Seven Interleukin-18(IL-18) variants were reported. Only three of them were considered.

IL-18_ rs1946518 (−607C/A). meta-analysis was completed with data of four publications (73/129/130/131). We didn’t find significant associations (OR 1.02; 95% CI 0.90–1.16) applying a fixed-effect model (Figure 5C), and publication bias wasn’t detected in funnel plot (*t* = 2.96; *p* = 0.098) (Supplementary Figure S3A).

IL-18 _ rs187238 (−137G/C). Three relevant studies were considered in this meta-analysis (129/130/131). We didn’t observe significant associations (OR 1.10; 95% CI 0.94–1.28) in a fixed-effect model (Figure 5D), and publication bias wasn’t seen (*t* = 2.96; *p* = 0.098) (Supplementary Figure S3B).

IL-18 _rs360721(C133/140G). Three studies (129/130/131) were involved in the meta-analysis. We didn’t observe significant associations (OR 0.89; 95% CI 0.77–1.04) applying the fixed-effect model (Figure 5E). No potential publication bias was identified (*t* = 10.68; *p* = 0.059) (Supplementary Figure S3C).

#### 3.2.6 The Tim-3 Gene

After screening, only one of the main allelic mutants of T cells immunoglobulin domain and mucin domain protein-3(Tim-3) were analyzed.

**FIGURE 6:**
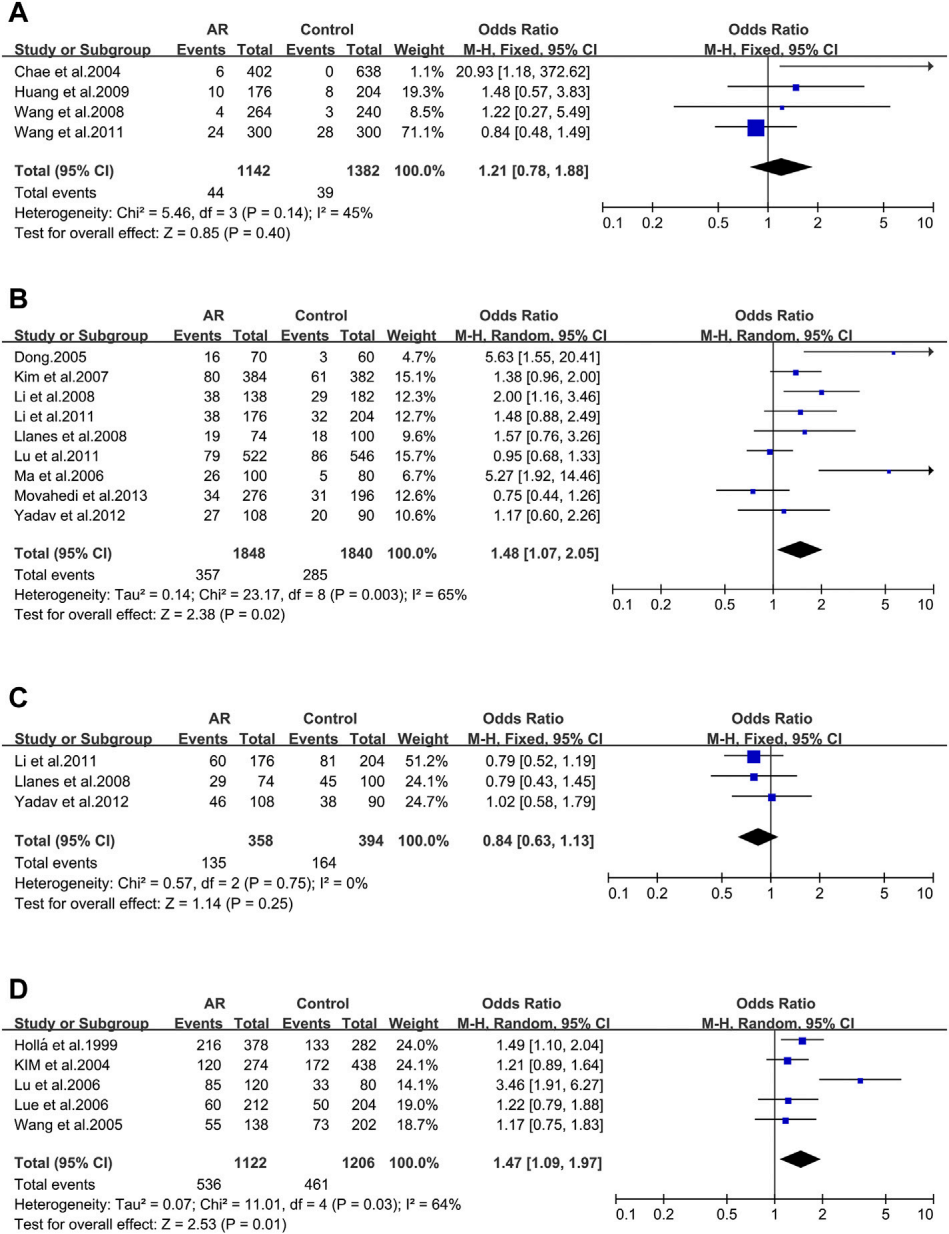
Forest plots for Tim-3, IL-4R and ACE polymorphisms. **(A)** Tim-3 (rs10515746): T allele; **(B)** IL-4R (rs1801275): allele R; **(C)** IL-4R (rs1805010): allele V; **(D)** ACE (I/D): allele D.

Tim-3_ rs10515746 (−574G/T). Four studies (6/44/49/132) were involved in this meta-analysis. We didn’t find significant associations (OR 1.21; 95% CI 0.78–1.88) in a fixed-effect model (Figure 6A), and publication bias wasn’t identified as well (*t* = 2.84; *p* = 0.105) (Supplementary Figure S3D).

#### 3.2.7 The IL-4R Gene

AFTER our data screening, both of the primary allelic mutants of IL-4R were analyzed.

IL-4R_ rs1801275 (Q576R/Gln551Arg). Nine studies (35/36/43/107/109/113/116/117/142) were considered in this meta-analysis. We observed significant associations (OR 1.48; 95% CI 1.07–2.05) applying a random-effect model (Figure 6B), and a publication bias across studies was detected (*t* = 2.62; *p* = 0.034) (Supplementary Figure S4A).

IL-4R_ rs1805010 (Ile50Val). Three studies (113/117/142) were included for this variant. No association was identified (OR 0.84; 95% CI 0.63–1.13) (Figure 6C) with no publication bias (*t* = 0.51; *p* = 0.699) (Supplementary Figure S4B).

#### 3.2.8 The ACE Gene

ACE_ I/D. When all the five studies were pooled together, we observed a significant association in a random-effect model (OR 1.47; 95% CI 1.09–1.97) (Figure 6D). And publication bias wasn’t identified (*t* = 1.25; *p* = 0.301) (Supplementary Figure S4C).

#### 3.2.9 The TAP1 Gene

**FIGURE 7:**
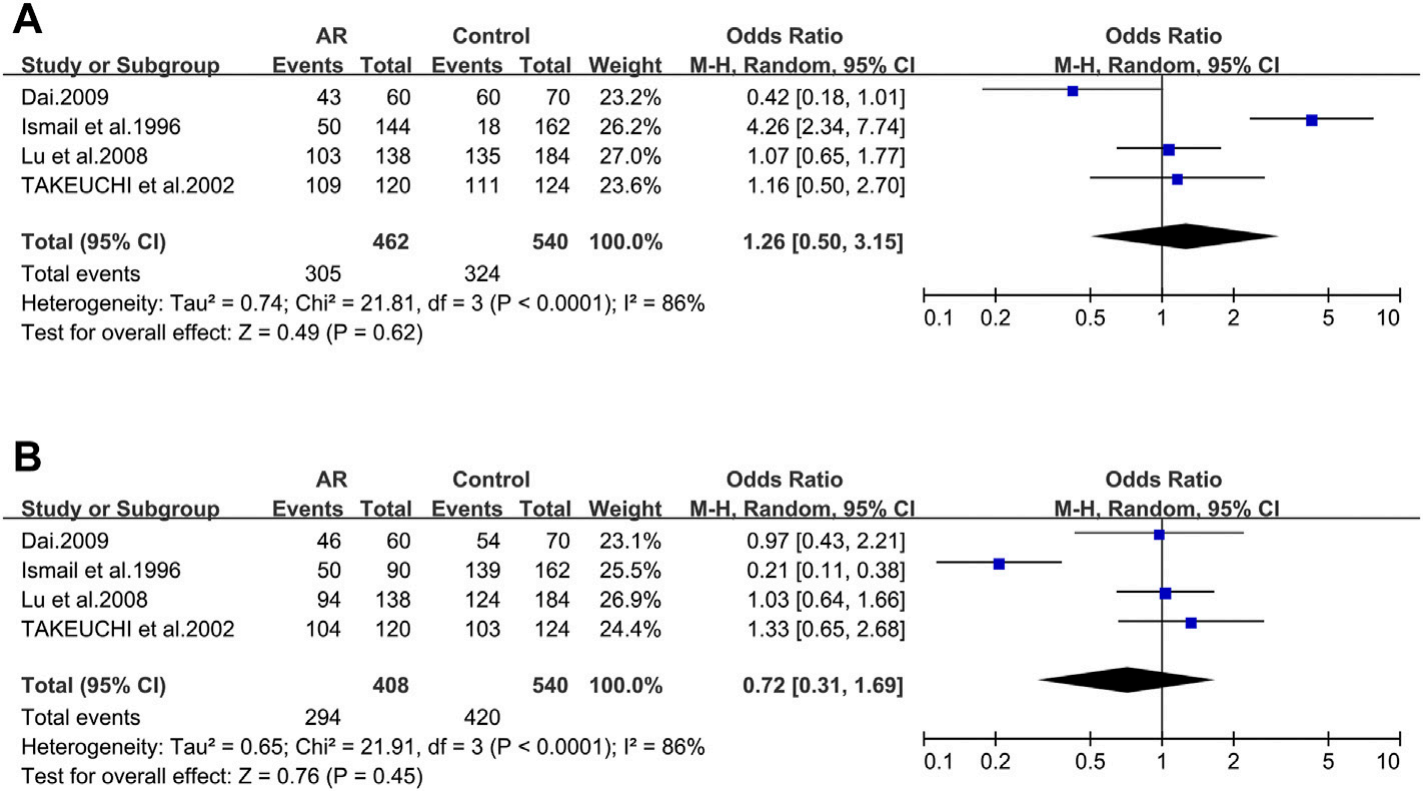
Forest plots for TAP1 polymorphisms. **(A)** TAP1 (333): I allele; **(B)** TAP1 (637): allele D.

TAP1_333V/I. Four studies dealing with ACE and AR were identified. Based on these four remaining studies (21/56/137/138), we didn’t find significant associations (OR 1.26; 95% CI 0.50–3.15) in a random-effect model (Figure 7A) and no potential publication bias was detected as well (*t* = −0.57; *p* = 0.627) (Supplementary Figure S4D).

TAP1_637G/D. Of the four selected studies for TAP1_637G/D (21/56/137/138), meta-analysis was completed with information of these publications, and we didn’t find significant associations (OR 0.72; 95% CI 0.31–1.69) employing a random-effect model (Figure 7B), as well as the publication bias (*t* = 0.00; *p* = 0.999) (Supplementary Figure S4E).

## 4 Discussion

The aim of this research was to offer a detailed review of polymorphisms about AR and to conduct a meta-analysis of those most frequently studied polymorphisms. Finally, 23 polymorphisms (in 12 genes) were meta-analyzed. Four polymorphisms were identified to be significantly related to the risk of AR. Thus, we were allowed to identify those polymorphisms that had the potential to constitute a screening test for AR and put forward combinations of SNPs that could prerecognize AR: IL-13 rs20541, CTLA-4 rs11571302, IL-4R rs1801275 and ACE (I/D).

To be as detailed as possible, we conducted extensive PubMed and CNKI search queries. The amount of 142 papers (involving 78 genes) were found to be related. To increase efficiency, only the genes that had been researched in three or more studies was considered in our study. Of the 142 publications cited in the Results section, 38 was augmented manually for the reason that the keyword “AR” was missing. The manual augment of references was not unexpected, because we were allowed to find relevant data quickly and accurately by our strategy.

To the best of our knowledge, our study is the first to have emphasized the potential association between AR and the CTLA-4 rs11571302 polymorphisms through meta-analysis (Figure 2C). The CTLA-4 gene plays a negative regulatory role in antigen-presenting cells (APC) activation of T cells (Walunas et al., 1996). Inducible CTLA-4 under-expression with Th2/Treg imbalance is associated to AR (Botturi et al., 2011). In our meta-analyses, CTLA-4 rs11571302 was related to AR (with OR of 1.41 [95% CI 1.24–1.60]). The frequencies of the rs11571302 A allele was obviously associated with the risk of AR. In contrast to the result for CTLA-4 rs11571302, the literature data on CTLA-4 rs3087243, CTLA-4 rs231725, and CTLA-4 rs11571315 show that they have no noticeable association with the AR risk.

Inflammation and immunization are known to be involved in the pathogenesis of AR. The Type 2 cytokines (IL-13, IL-4 and IL-18) are the initiating factors of airway inflammatory response (Wan et al., 2015; Che et al., 2018). Several studies have unveiled that levels of IL-13, IL4 and IL18 in peripheral secretion appear to be abnormally high in patients with AR (Shamji et al., 2021). Our meta-analysis emphasized an association between the IL-13 rs20541 polymorphism and AR risk. IL-13 rs20541 imparts susceptibility to the development of AR. However, we observed no link between IL-13 rs1800925 and AR. These results were similar to the previous meta-analyses (Chen et al., 2018; Tang et al., 2020).

No associations with AR were detected here for IL-4 rs2243250, IL-4 rs2227284 and IL-4 rs2070874. In contrast, one previous study reported that the IL-4 rs2243250 genotype is associated with AR susceptibility (Jiang and Yan, 2021). We excluded one study not published online any more which was taken into account in the previous meta-analysis. Obviously, the selection of control participants affects the final result. Considering the instability of the results, further studies are required to get a more comprehensive result to ensure or refute the underlying association between AR and the IL-4 rs2243250 polymorphism. Associations were not also found for IL-18 rs1946518, IL-18 rs187238 and IL-18 rs360721, which confirmed with the earlier meta-analysis (Tang et al., 2020). Further researches are deemed unnecessary.

All biological functions of IL-4 are mediated by effector IL-4R, including mediating Th1/Th2 imbalance, promoting B cell proliferation and IgE synthesis, which play a crucial role in allergic rhinitis (Moniuszko et al., 2013). Our result exhibited that IL-4R rs1801275 was associated with the risk of AR (OR 1.48; 95% CI 1.07–2.05), and the R allele increase the susceptibility to AR. But the correlation needs to be validated in more studies, because there is potential publication bias in Egger’s test. In contrast, we detected no association between AR and IL-4R rs1805010 polymorphism.

ACE I/D polymorphism may influence the strength of immunological response and has been reported to be associated with atopic disorders, inflammatory diseases (Eryüksel et al., 2009). Our result revealed that ACE D allele augments the risk of developing AR, concordant with previous researches (Huang et al., 2016; Li et al., 2017).

TNF-α functions as a pro-inflammatory cytokine involving in T cell activation and cytokine release, recruitment of neutrophils, macrophages and monocytes, and increased histamine release from airway mast cells. Here, we ensured the known result of association previously shown by another meta-analysis (Zhou, 2019). We found no correlation between AR and TNF-α (−308G/A).

TGF-β is known to be involved in AR, which can induce initial CD4 + T cells to differentiate into CD4 + CD25 + Treg cells and stabilize the immune function of the body (Gu et al., 2017). Elevated levels of TGF-β1 can alleviate the inflammatory of nasal mucosa in AR (Peng et al., 2022). To date, the results focused on the association between TGF-β1 rs1800469 polymorphism and AR risk were inconsistent. One paper have demonstrated that TGF-β1 polymorphism has a protective effect for AR (Liu et al., 2009). Conversely, the results from other researches shown that the TGF-β1 SNP could increase the susceptibility of developing AR or was not associated with AR (Qiu et al., 2009; Cárdaba et al., 2014; Zhu et al., 2020). Our present work didn’t find a relation of AR and TGF-β1 rs1800469 polymorphism. Additional well-designed case-control researches in large cohorts are needed to confirm the underlying link between AR and TGF-β1 rs1800469 polymorphism.

Studies have shown that CD14/-159 may influence the regulation of CD14 gene expression and thus modulate the influence of CD14-mediated events on innate and adaptive immune responses (LeVan et al., 2001). CD14 specifically binds to LPS to support the conversion of Th0 to Th1, and cytokines produced by Th1 and Th2 control the synthesis of IgE (Baldini et al., 1999). Compared with normal people, the serum CD14 in AR patients is significantly higher, and the risk of AR is significantly associated with the homozygous carriers of the T allele (TT genotype) (Zare Marzouni et al., 2019). Interestingly, it was reported that TT genotype carriers have a significant increase in serum levels of sCD14 and a concomitant decrease in total serum IgE (Qiu et al., 2009). However, TT homozygotes expression was higher in AR patients, but not associated with IgE expression among the Chinese population (Han et al., 2010). In this meta-analysis, CD14 (−159C/T) T allele was not associated with AR susceptibility, which was in line with previous studies (Xu and Wang, 2014; Chen et al., 2018).

FOXP3, as a specific transcription factor of Treg cells, is involved in the development and functional maintenance of Treg cells (Hori et al., 2003). Compared with healthy people, the expression of FOXP3 in peripheral blood of AR patients is significantly decreased, which leads to the dysfunction of Treg cells and is an important factor leading to the onset of AR (Huang et al., 2014). Our result was similar to the earlier meta-analysis (Zhang et al., 2017; Tang et al., 2020). There was no link between AR and FOXP3 rs3761548 or FOXP3 rs2232365.

Tim-3 has a negative regulatory effect on Th1 cell and causes Th1/Th2 imbalance, which is required for the development of AR (Pan et al., 2010). It was reported that increased level of Tim-3 in the peripheral blood of AR patients (Shi and Liu, 2018). However, the meta-analysis result of Tim-3 rs10515746 indicated that it was not significantly related to the AR risk.

Inhaled antigen is presented to CD4 + T cells in AR, and TAP1 gene encodes molecules involved in endogenous antigen processing (Kim et al., 2007). TAP gene polymorphism may affect antigenic peptide selection and transport processes and change immune response regulation in AR. However, we did not identify the potential link between AR and TAP1 333V/I or TAP1 637G/D.

In the end, this study has several limitations. Firstly, for greater efficiency, it only considered the polymorphisms cited in more than three various studies, which may miss some significant associations to AR. Thus, to confirm the underlying correlations between the remaining polymorphisms and the susceptibility of AR, more studies will be necessary. Second, the susceptibility to AR is not only related to heredity, but also to race, age, sex and so on. However, due to the small number of studies and small samples in the present analysis, stratifications for race, age and sex were not performed. Besides, the susceptibility to AR may be affected by multiple gene loci, which coordinate with each other to offset the genetic susceptibility to developing AR. These may be the reasons why only 4 SNPs were observed to have an association with the risk of AR in this study.

## 5 Conclusion

As mentioned above, four of the polymorphisms could be analyzed simultaneously as they were associated with AR. For the few polymorphisms not involved in this study, that is, those showing a positive correlation based on two studies, further research is needed. This work allow researchers begin to find the underlying markers for developing a diagnostic method for AR patients. The panel of these polymorphisms exhibits the potential in forming a genetic screening test about AR and may thus lessen the time used in diagnosing AR.

## Data Availability

The original contributions presented in the study are included in the article/Supplementary Material, further inquiries can be directed to the corresponding author.
