# Supplementary material for: Polymorphisms and AR: A Systematic Review and Meta-Analyses
Source: Front Genet. 2022 Jul 1;13:899923. doi: 10.3389/fgene.2022.899923 (PMC9284009; doi:10.3389/fgene.2022.899923)
Supplement: Supplementary file 3 [file DataSheet1.PDF]

Supplementary Material

Supplementary Figures

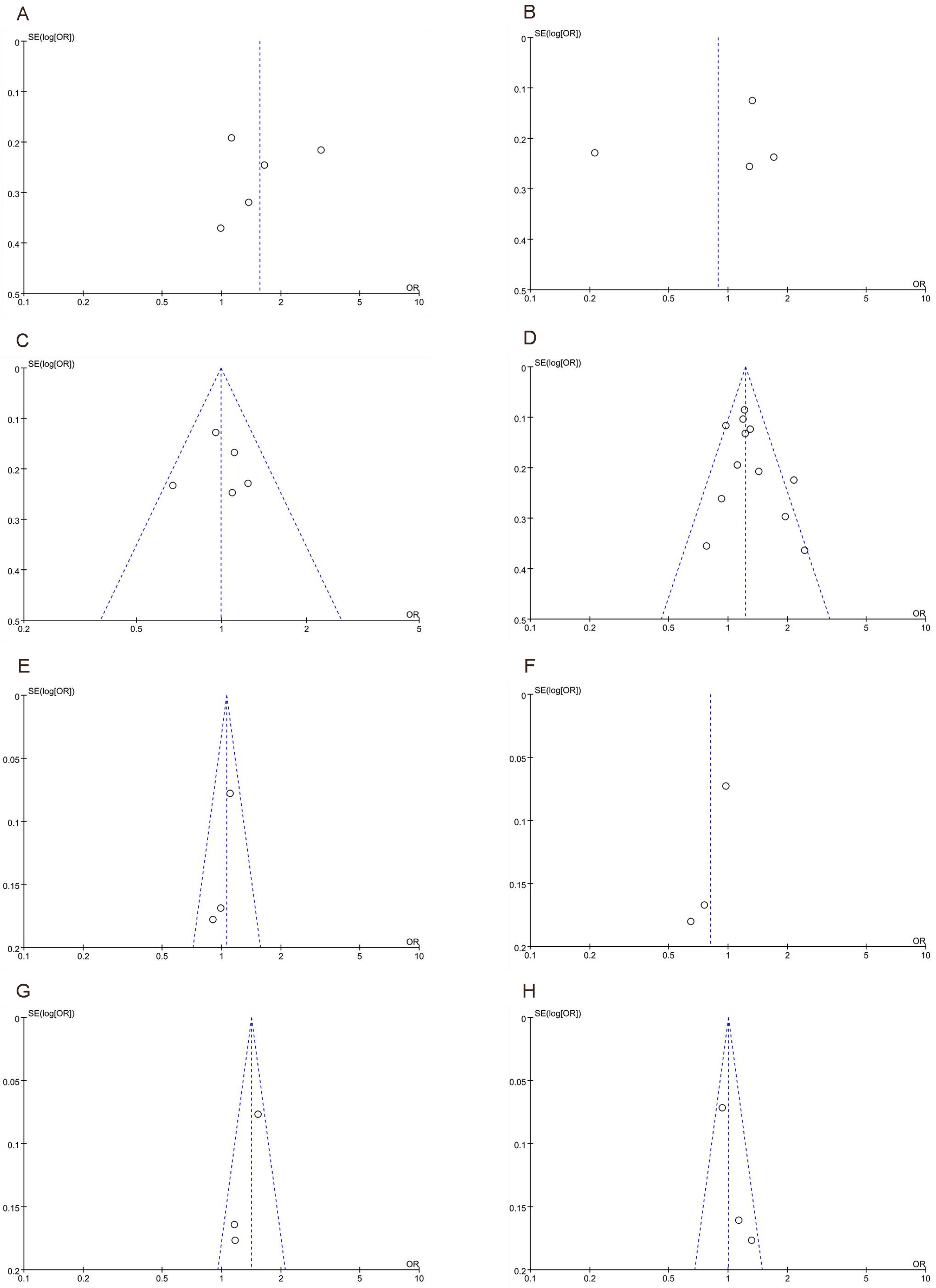

Supplementary Figure 1. Funnel plot of (A) TNFα(rs1800629); (B) TGFβ1(rs1800469); (C) IL-13(rs1800925); (D) IL-13 (rs20541); (E) CTLA-4 (rs3087243); (F) CTLA-4 (rs231725); (G) CTLA-4(rs11571302); (H) CTLA-4(rs11571315).

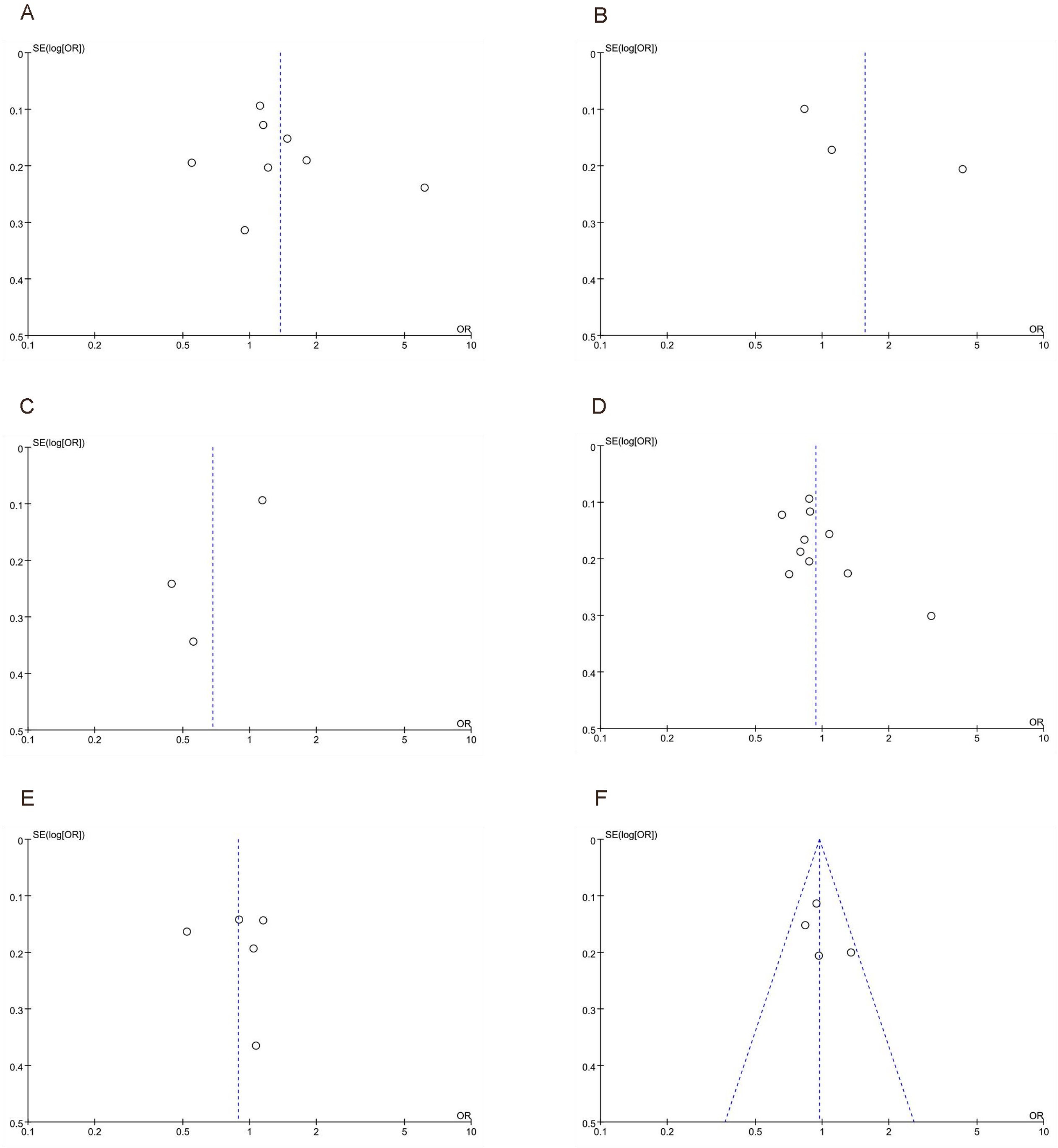

Supplementary Figure 2. Funnel plot of (A) IL-4(rs2243250); (B) IL-4(rs2227284); (C) IL-4(rs2070874); (D) CD14 (rs2569190; (E) FOXP3(rs3761548); (F) FOXP3(rs2232365).

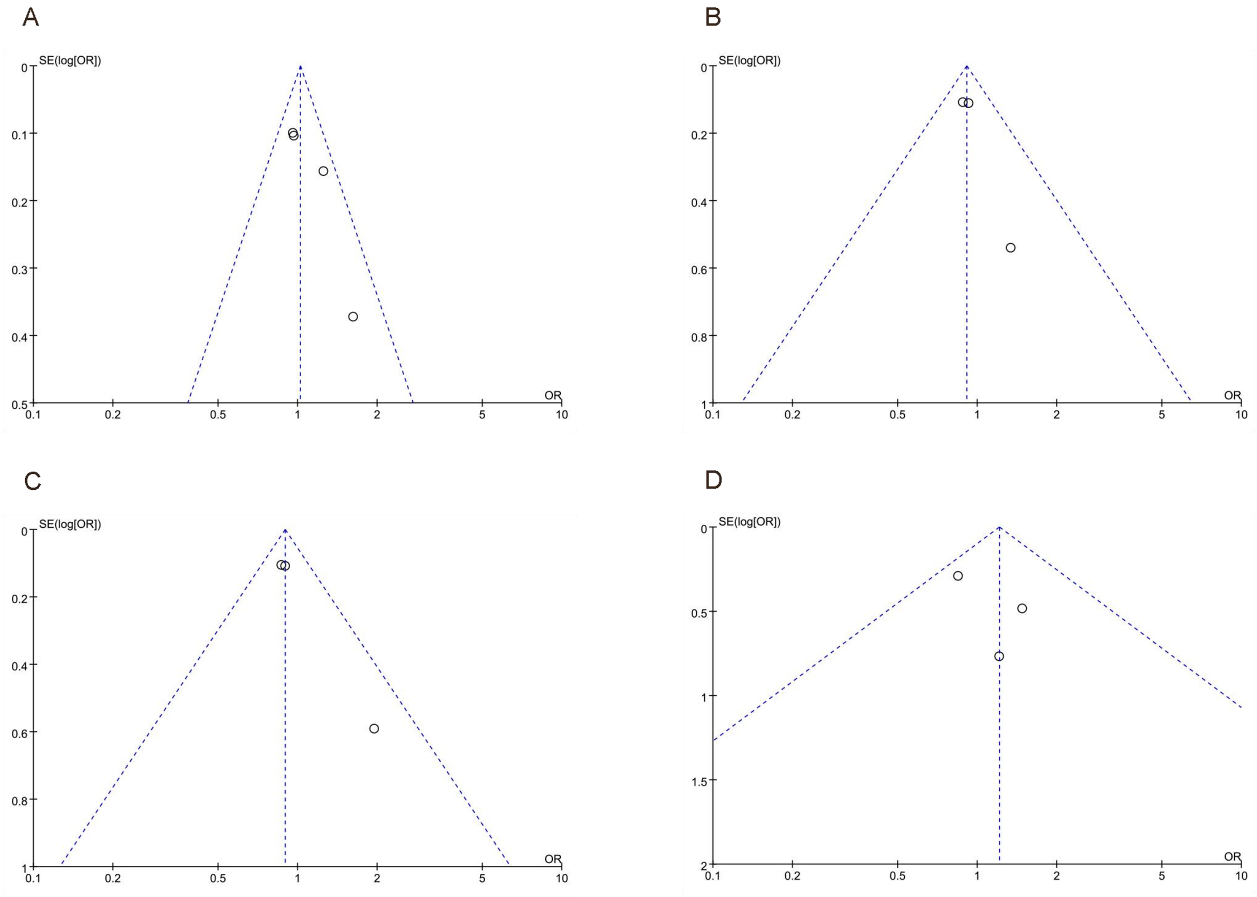

Supplementary Figure 3. Funnel plot of (A) IL-18 rs1946518); (B) IL-18 (rs187238); (C) IL-18 (rs4988359); (D) Tim-3 (rs10515746).

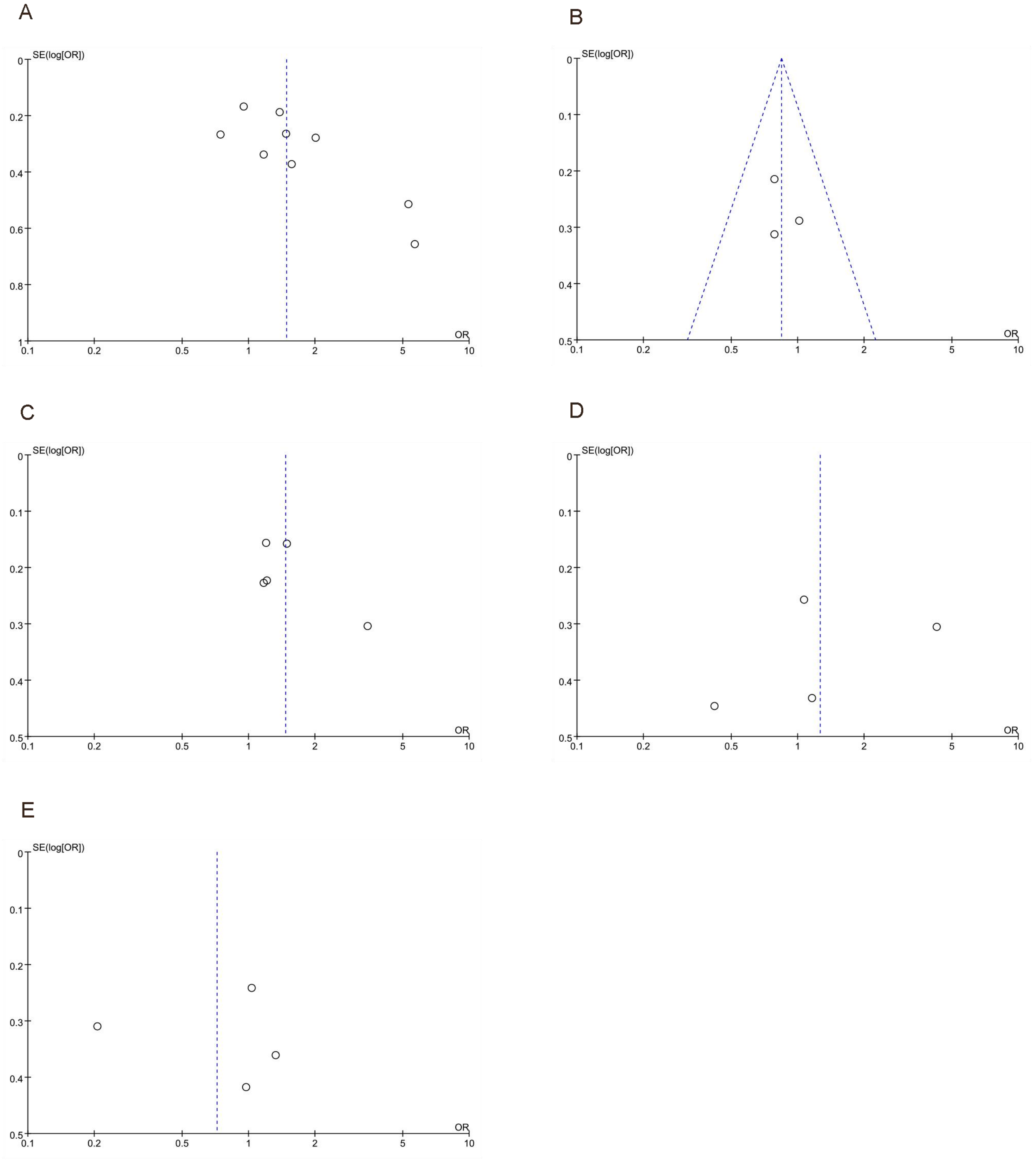

Supplementary Figure 4. Funnel plot of (A) IL-4R (rs1801275); (B) IL-4R (rs1805010); (C) ACE (I/D); (D) TAP1 (333); (E) TAP1 (637).
